# Molecular and Evolutionary Mechanisms of Cuticular Wax for Plant Drought Tolerance

**DOI:** 10.3389/fpls.2017.00621

**Published:** 2017-04-28

**Authors:** Dawei Xue, Xiaoqin Zhang, Xueli Lu, Guang Chen, Zhong-Hua Chen

**Affiliations:** ^1^College of Life and Environmental Sciences, Hangzhou Normal UniversityHangzhou, China; ^2^College of Agriculture and Biotechnology, Zhejiang UniversityHangzhou, China; ^3^School of Science and Health, Hawkesbury Institute for the Environment, Western Sydney University, RichmondNSW, Australia

**Keywords:** drought stress, cuticular wax composition, cuticular wax biosynthesis, gene expression, comparative genomics

## Abstract

Cuticular wax, the first protective layer of above ground tissues of many plant species, is a key evolutionary innovation in plants. Cuticular wax safeguards the evolution from certain green algae to flowering plants and the diversification of plant taxa during the eras of dry and adverse terrestrial living conditions and global climate changes. Cuticular wax plays significant roles in plant abiotic and biotic stress tolerance and has been implicated in defense mechanisms against excessive ultraviolet radiation, high temperature, bacterial and fungal pathogens, insects, high salinity, and low temperature. Drought, a major type of abiotic stress, poses huge threats to global food security and health of terrestrial ecosystem by limiting plant growth and crop productivity. The composition, biochemistry, structure, biosynthesis, and transport of plant cuticular wax have been reviewed extensively. However, the molecular and evolutionary mechanisms of cuticular wax in plants in response to drought stress are still lacking. In this review, we focus on potential mechanisms, from evolutionary, molecular, and physiological aspects, that control cuticular wax and its roles in plant drought tolerance. We also raise key research questions and propose important directions to be resolved in the future, leading to potential applications of cuticular wax for water use efficiency in agricultural and environmental sustainability.

## Introduction

Abiotic stresses such as light, drought, salinity, heat, cold, waterlogging, nutrient deficiency, and other non-biotic environmental conditions (Chen et al., 2005; Honsbein et al., 2009; Wang et al., 2013, 2016; Dai et al., 2014; Liu et al., 2014; Mak et al., 2014; O’Carrigan et al., 2014) are the major limiting factors, affecting plant growth and development, and frequently result in reduced crop productivity (Fang and Xiong, 2015; Joshi et al., 2016). As a major cause affecting plant growth and productivity, drought stress reduces crop yield worldwide (Pennisi, 2008; Zhang M. et al., 2015). Globally, the losses of crop yield to drought are estimated to be up to 30% by 2025 in comparison to current yield (Zhang, 2011).

Unlike animals, plants must withstand abiotic stresses at the sites of growth. To survive under drought stress, plants have evolved comprehensive mechanisms through integrated molecular and cellular responses to achieve physical adaptations such as deep root system (Comas et al., 2013), efficient stomatal structure and regulation (Chen et al., 2017; Pornsiriwong et al., 2017), leaf morphology (Farooq et al., 2009), cuiticular wax thickening (Shepherd and Wynne Griffiths, 2006) and cutinization of the leaf surface (Shao et al., 2008). Among these physical adaptations, cuticular wax provides an essential barrier to protect plants from drought stress (Samuels et al., 2008; Lee and Suh, 2015a). As a major component of cuticle, cuticular wax is the outermost hydrophobic layer, serving as a barrier to restrain uncontrolled non-stomatal plant gas exchange. Also, it protects plants against high temperature, strong UV radiation, bacterial and fungal pathogens as well as insects, increases plants’ tolerance to high salinity and low temperature (Domínguez et al., 2011; Yeats and Rose, 2013; Lee and Suh, 2015a). In addition, it was also found that cuticular wax is involved in the processes of plant morphology and development through tight epidermal connections (Javelle et al., 2011).

The composition, biochemistry, structure, biosynthesis, and transport of plant cuticular wax have been reviewed extensively. The readers are directed to some excellent reviews (Kunst and Samuels, 2003; Bernard and Joubès, 2013; Yeats and Rose, 2013; Lee and Suh, 2015a; Fernández et al., 2016; Fich et al., 2016). However, there are still significant gaps in molecular mechanisms of plant cuticular wax involving in drought stress response. In the present review, we focus on molecular, evolutionary, and physiological mechanisms that control plant cuticular wax changes in response to drought stress.

## Evolution of Plant Cuticular Wax

More than 450 million years ago, plants evolved into a terrestrial lifestyle from aquatic environments. The colonization of plants on land is one of the most important events in life evolution, with far-reaching consequences to shape the global ecosystem. Compared to aquatic plants and algae, terrestrial plants have to face larger frequency of desiccation, more fluctuating temperatures, and higher solar radiation, so the first land plants need to change their developmental and morphological mechanisms to adapt to their new life on land (Graham, 1993). One of the most essential adaptive traits for terrestrial living of plants would have been their capacity to efficiently maintain water in a dry habitat via many adaptive strategies and their evolution. To adapt to the terrestrial environment of water shortage, terrestrial plants have developed a unique structure on the surface of aerial organs called cuticle (Kenrick and Crane, 1997; Yeats and Rose, 2013). The evolutionary innovation of cuticle is a milestone in plant evolution due to its universal importance.

The importance of curticular wax in plant drought tolerance is evident that, compared to gymnosperms and angiosperms, many early extant plants such as liverworts, mosses, lycophytes, ferns, and horsetails are relatively more sensitive to drought (Edwards et al., 1996). Many species in these taxa are therefore limited in shade and wet growth habitats (Jeffree, 2006). Do these differences in drought sensitivity relate to the occurrence, structure, synthesis, composition, and molecular regulation of cuticular wax in diverse plant taxa? Growing evidences suggest that cuticular wax is important in maintaining plant water status in various species and mutants (Jetter et al., 2006; Shepherd and Wynne Griffiths, 2006; Yeats and Rose, 2013; Lee and Suh, 2015a). Although no stomata are available for active regulation of transpiration in one of the most ancient extant plants – liverworts (Chen et al., 2017), a hydrophobic cuticle was found at the surface of the liverworts analogous to a procuticle (Cook and Graham, 1998). In moss, the structure of cuticles parallels that of vascular plants with the major composition being hexadecanoic acid (Caldicott and Eglinton, 1976). ω-hydroxymonobasic acids and 10,16-dihydroxyhexadecanoic acid are major components of the cutins of fern and lycophyte (Hunneman and Eglinton, 1972; Caldicott and Eglinton, 1976), similar to those of cutins in angiosperms and gymnosperms (Holloway, 1982). In angiosperms, especially crop species, it was found that the leaves’ water retention ability was increased largely due to the deposition of more leaf surface wax and the higher internal tissue lipid accumulation of the drought tolerant forage crops (Saneoka and Ogata, 1987). The cuticular wax load increased significantly and correlated significantly with harvest index when subjected to drought treatment in pea plants (Sánchez et al., 2001). The *gl1-1/wsl2* and *gl1-2* loss-of-function rice mutants with reduced wax load exhibited higher sensitivity to drought in contrast to the wild type (WT) plants (Islam et al., 2009; Qin et al., 2011; Mao et al., 2012).

Cuticular wax is reported to occur on the surfaces of all tested land plants (Jetter et al., 2006). The ultrastructure of the cuticular wax of many species is both complex and variable. Compounds forming cuticular wax in mosses and liverworts were identical to those of gymnosperms and angiosperms, suggesting that cuticular wax evolved in the early stages of terrestrial plant evolution (Jetter et al., 2006). Molecular analysis of wax-deficient mutants such as *eceriferum* (*cer*), *bloomless* (*bm*), and *glossy* (*gl*) has led to the identification of large number of genes encoding functional proteins in the biosynthesis, transport, and regulation of cuticular wax in *Arabidopsis thaliana*, *Eutrema salsugineum*, *Zea mays*, *Oryza sativa*, *Triticum aestivum*, *Lycopersicon esculentum*, *Petunia hybrida*, *Medicago sativa*, *Medicago truncatula*, *Brassica campestris*, and *Camelina sativa* (Lee and Suh, 2015a). However, few genes were reported for the biosynthesis, transport and regulation of cuticular wax in non-flowering plants (Buda et al., 2013). Therefore, a better understanding of molecular evolution of cuticular wax related genes in terrestrial plants requires further study on those early lineages of plant species.

## Structure and Composition of Plant Cuticular Wax

Cuticle widely distributes on plant surface, occurring in shoots, radicles, fruits, flowers, and leaves (Riederer and Schreiber, 2001). Cuticle consist cutin polyester matrix and intracuticular and epicuticular waxes to form a hydrophobic surface for the protection of plants (Samuels et al., 2008; Nawrath et al., 2013; Yeats and Rose, 2013). Cuticular wax is one major constituent dispersed across the entire depth of the cuticle (Lee and Suh, 2015a). Cuticular wax often forms complex microstructures with three dimensional crystalline. Generally, plant cuticular wax has two physical layers: ‘intracuticular wax’ and ‘epicuticular wax.’ The former is dispersed in the cutin polymer and the latter is deposited on the outer surface (Jeffree, 2006; Buschhaus and Jetter, 2011). It is well-recognized that plant cuticular waxes are organic solvent-extractable complex mixtures of hydrophobic lipids, consisting mostly of very-long-chain fatty acids (VLCFAs) and their derivatives. These VLCFAs include, alkanes, wax esters, branched alkanes, primary alcohols, alkenes, secondary alcohols, aldehydes ketones, and unsaturated fatty alcohols, as well as cyclic compounds including terpenoids and metabolites such as sterols and flavonoids (Jetter et al., 2006; Samuels et al., 2008; Lee and Suh, 2013, 2015a; Yeats and Rose, 2013).

There are a great variety of structure and composition of cuticular waxes among different plant species as well as in different tissues and organs (Jetter et al., 2006; Buschhaus and Jetter, 2011; Bernard and Joubès, 2013). There are even distinctive cuticular waxes in different growth and developmental stages. Many environmental factors (e.g., light, temperature, and humidity) also influence wax composition considerably in the same species (Geyer and Schönherr, 1990; Kolattukudy, 1996; Knight et al., 2004; Samuels et al., 2008). These results suggest novel genes involved in cuticular wax biosynthesis may be practically used as valuable genetic resource to improve crop drought tolerance in plant breeding. However, future studies should be conducted to illustrate the main factors affecting loads and compositions of the diversity and response of cuticular wax to drought stress.

## Cuticular Wax Biosynthesis and Transport

**FIGURE 1:**
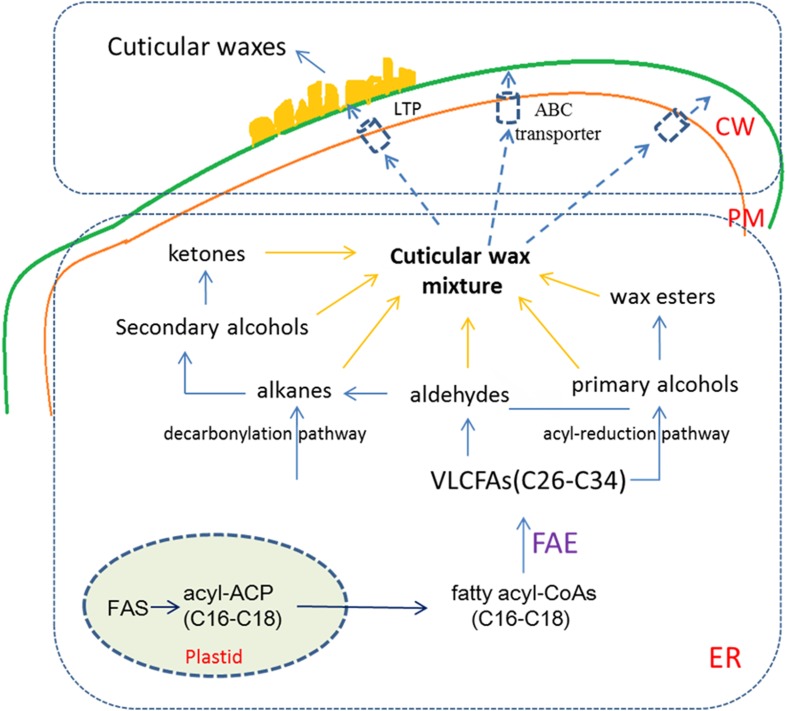
**The biosynthesis and transport of plant cuticular wax**. In epidermal cells, C_16_ and C_18_ fatty acids are synthesized in the plastid, then C_16_ and C_18_ fatty acyl-CoAs are elongated to VLCFAs (C_26_ to C_34_) in the ER. VLCFAs are modified according to two distinct biosynthetic pathways to generate the aliphatic compounds of waxes. In the interaction of ABC transporter and LTP, wax mixture are mobilized from the ER to the PM, and then exported from the PM to the extracellular matrix. CW, cell wall; ER, endoplasmic reticulum; PM, plasma membrane; FAE, fatty acid elongase; ABC transporter, ATP binding cassette transporter; LTP, lipid transfer protein.

The biosynthesis of cuticular wax is a complex process (**Figure 1**). Cuticular wax is synthesized on the outer membrane in the plastid of epidermal cells with the *de novo* C_16_ and C_18_ fatty acid synthesis. C_16_ and C_18_ fatty acids serve as central intermediates for lipid classes. In the endoplasmic reticulum (ER), fatty acyl-CoAs (C_16_ and C_18_) are elongated to wax precursors of VLCFAs with C_26_ to C_34_ chains by a repeating reaction process via fatty acid elongase (FAE) complex. Following elongation, wax components are finally produced by converting long-chain fatty acyl-CoAs via two pathways. The acyl-reduction pathway generates primary alcohols and wax esters and the decarbonylation pathway, produces alkanes, aldehydes, secondary alcohols, and ketones. As major wax components found in a wide range of plant species, biosynthesis of primary alcohols is completed by acyl-reduction pathway, in which fatty acyl-CoA reductase (FAR) converts fatty acyl-CoAs into primary alcohols (Samuels et al., 2008; Kunst and Samuels, 2009).

Components of plant cuticular wax synthesized in the ER need to be transported to the cuticle. Firstly, the components are transferred from the ER to the plasma membrane (PM), then exported to the apoplast across the PM, and transported to the plant surface via the cell wall (Samuels et al., 2008; Yeats and Rose, 2013; Lee and Suh, 2015a). Cuticular wax export is mediated by the ATP binding cassette (ABC) transporters. Using mutants with reduced cuticular wax content, *CER5/ABCG12* and *ABCG11* genes were cloned and found that these genes encode members of the ABC transporters (Pighin et al., 2004; Bird et al., 2007; Panikashvili et al., 2007). Members of *LTPG (lipid transfer protein)* gene family *LTPG1* and *LTPG2* have been shown to participate in the output of cuticular wax (DeBono et al., 2009; Kim et al., 2012; Albert et al., 2013). However, whether LTPG has the ability to penetrate the cell wall for lipid secretion into the epidermis remains to be verified (DeBono et al., 2009; Lee et al., 2009). Recently, a new *Arabidopsis* acyl-CoA-binding protein (AtACBP1) was identified to be localized in the ER and the PM. *AtACBP1* is mainly involved in the secretion of lipid components and stem cuticle formation (Xue et al., 2014). Some studies have shown that a number of proteins, which are encoded by the genes responsible for cuticular wax biosynthesis such as *AtCER1*, *AtCER4*, *AtWAX2, At5g02890, TaFAR1, OsWS1* and *CsWAX2*, were found to be localized on the ER (Kamigaki et al., 2009; Wang et al., 2015, 2016; Xia et al., 2015; Xu et al., 2017). These results showed that the ER is an important site for the synthesis of cuticular wax in plants.

In recent years, a number of genes related to cuticular wax biosynthesis and transport have been characterized from plants through biochemical, genetic, genomic, molecular, and cell biological approaches, and the recent advances have been reviewed comprehensively elsewhere (Samuels et al., 2008; Yeats and Rose, 2013; Lee and Suh, 2015a).

## Linking Cuticular Wax to Plant Drought Tolerance

Plant cuticular waxes play important role as a protective barrier against environmental stresses, and their biosynthesis, transport, deposits, and composition, also were affected by various environmental factors (Shepherd and Wynne Griffiths, 2006). For instance, one of the most important functions for cuticular wax is to protect plants from excessive ultraviolet (UV) light. Many studies showed that elevated UV-B radiation affects plant cuticular wax formation and gas exchange (Jansen et al., 1998; Fukuda et al., 2008; Jiang et al., 2009). It was found that cuticular wax content increases by 20–28% in cucumber (*Cucumis sativus* L.), bean (*Phaseolus vulgaris* L.), and barley (*Hordeum vulgare* L.) when plants are exposed to UV-B light (Steinmüller and Tevini, 1985). It was reported that increasing gas exchange rate for *in vitro* grown carnation plants leads to thicker epidermal wax (Majada et al., 2001), and larger amount of wax covering the leaf surface affect rate of gas exchange in mulberry (*Morus alba*) (Yu et al., 2015) and canola (*Brassica napus*) (Ni et al., 2014). However, the regulatory mechanism of changes of cuticular wax induced by high UV-B radiation and its link to gas exchange and plant performance needs further study. Here, we only discuss the current advances and the roles of cuticular wax in plant drought tolerance.

Many studies have shown that drought increases the content and changes composition of cuticular wax in *Arabidopsis* (Aharoni et al., 2004; Kosma et al., 2009; Seo et al., 2011; Lee and Suh, 2015a), wheat (Zhang et al., 2013; Zhang Z. et al., 2015), pea (Sánchez et al., 2001), alfalfa (Zhang et al., 2005), rose (Jenks et al., 2001), and tobacco (Cameron et al., 2006). The total amount of wax per unit leaf area increased by 80% in plants under water deficit than those in the control, and the water deficit-treated plants had 49% increases in cuticle thickness. In general, alkanes, attributed to the increase in constituents of the long chains (C_29_, C_31_, and C_33_), accounted for the observed 93% increases in total wax amount, in plants under water deficit (Kosma et al., 2009). Jenks et al. (2001) found the wax load per unit leaf area increased and the proportions of individual wax constituents also change in roses subjected to periods of moderate drought during production. In sorghum, compared to a bloomless line with 15% lower amount of wax on the epidermal surface, the cuticular resistance (as a component adaptation of plants to drought prone regions) (Moreshet, 1970 and equations therein) of a high wax accumulating bloom lines increased by 90% under in drought stress (Saneoka and Ogata, 1987). In rice, mutations in *wilted dwarf and lethal 1* (*WDL1*) influenced the crystallization and distribution of wax on the surface of leaf. The leaf surface of *wdl1* mutant was covered by disorganized and normal crystal waxes, including curdled wax, split wax cluster, and stretched wax curd. These increase its transpiration rates to 2.3 times higher than those in the WT, leading to increased rates of water loss and lower water use efficiency (WUE) (Park et al., 2010). It was reported that the thicker the wax layer the better the drought tolerance, however, many studies found that the thickness of wax layer is not directly related to water loss (Schreiber and Riederer, 1996; Sánchez et al., 2001; Vogg et al., 2004). Reduce of wax monomers especially C_29_ alkanes was considered to decline the tolerance of drought stress (Panikashvili et al., 2007). Kosma et al. (2009) also found that increases in the very-long-chain constituents lead to the change of the total amount of leaf wax after drought treatment. It is therefore speculated that alkane synthesis is key to drought stress response.

In tomato (*Lycopersicon esculentum*) plants, it was shown that the main transpiration barrier is located in the intracuticular wax layer and is mainly determined by the aliphatic constituents. This suggests that, instead of the cutin matrix, cuticular wax is responsible for the reduced non-stomatal transpiration (Vogg et al., 2004). Physiological analysis of an irradiated bloomless *KFS2021* sorghum mutant showed that it had higher rate of seedling water loss than the bloom WT, indicating that epicuticular wax may improve WUE by regulating water loss at night in sorghum (Burow et al., 2008). Furthermore, drought stress increased cuticular wax deposition by up to 2.5-fold in leaves of numbers of plant species including maize (*Zea mays*), wheat (*Triticum aestivum*), soybean (*Glycine max*), pine (*Pinus palustris*), oats (*Avena sativa*), tree tobacco (*Nicotiana glauca*), cotton (*Gossypium hirsutum*), and sesame (*Sesamum indicum*) (Cameron et al., 2006; Shepherd and Wynne Griffiths, 2006; Kosma and Jenks, 2007).

Cuticular wax also plays an important role in crop yield, and the increase of wax content are associated with enhanced drought tolerance in many plants. The drought-tolerance and yield were higher in crops having more cuticular wax than those with less wax or non-waxy crops (Zhou et al., 2013; Guo et al., 2016). There were significant corrections between the wax content and yield, drought tolerance and WUE in crops such as sorghum (Jordan et al., 1984), barley (Febrero et al., 1998), rice (Zhu and Xiong, 2013), and wheat (Richards et al., 1986). Johnson et al. (1983) found that the glaucous wheat genotypes have significantly more grain yield than non-glaucous ones in the normal and moderate drought environment. Moreover, field physiological studies found that wheat yield is positively related to cuticular wax, especially under drought conditions (Monneveux et al., 2004; Zhang et al., 2013). Similarly, the positive correlation between barley grain yield and the epicuticular wax load under drought, indicating drought tolerance of in these breeding lines (González and Ayerbe, 2010).

It is clear that cuticular wax protects plants during periodic drying and drought stress. However, it still needs comprehensive investigations, especially in the physiological and agronomical interrelationships between cuticular wax chemical structure, composition, and drought stress.

## Biosynthesis of Plant Cuticular Wax in Drought Stress Tolerance

Under drought stress, plants will execute a series of reactions, including abscisic acid (ABA)-induced stomatal closure, accumulation of cuticular wax and formation of deep root system, which in turn will further improve the drought tolerance of plants. During development, cuticular wax is deposited on the surface of plant and the total amount is regulated in response to the severity of drought, which is eventually regulated by ‘waxy’ genes. Increasing numbers of ‘waxy’ genes are cloned and their functions are being deciphered. Genes such as *eceriferum* (*CERs*), transcription factor (TF) *Wax Inducer1/SHINE1* (WIN1/SHN1), and *WAX2* (**Table 1**), have been proven and practical applied, especially in improving drought tolerance and adaptation of certain crops (Bernard and Joubès, 2013; Yeats and Rose, 2013; Lee and Suh, 2015a).

**Table 1:** Genes known to be involved in cuticular wax response to drought stress.

Gene name	Species	Function	Reference
*CER1*	*A. thaliana*	Aldehyde decarbonylase	Aarts et al., 1995; Bourdenx et al., 2011
*CER6*	*A. thaliana*	3-Ketoacyl-CoA synthase	Hooker et al., 2002; Karaba, 2007
*WAX2*	*A. thaliana*	Aldehyde decarbonylase	Chen et al., 2003; Karaba, 2007
*WIN1/SHN1*	*A. thaliana*	AP2/EREBP transcription factors	Aharoni et al., 2004; Broun et al., 2004
*SHN2*	*A. thaliana*	AP2/EREBP transcription factors	Aharoni et al., 2004
*SHN3*	*A. thaliana*	AP2/EREBP transcription factors	Aharoni et al., 2004
*WXP1*	*M. truncatula*	AP2 domain-containing putative transcription factor	Zhang et al., 2005
*OsWRKY89*	*O. sativa*	WRKY transcription factors	Wang et al., 2007
*LeCER6*	*L. esculentum*	3-Ketoacyl-CoA synthase	Leide et al., 2007
*WXP2*	*M. truncatula*	AP2 domain-containing putative transcription factor	Zhang et al., 2007
*OsWSL1*	*O. sativa*	3-Ketoacyl-CoA synthase	Yu et al., 2008
*OsGL1-1*	*O. sativa*	Aldehyde decarbonylase	Qin et al., 2011
*MYB96*	*A. thaliana*	MYB transcription factors	Seo et al., 2011
*OsGL1-2*	*O. sativa*	Fatty acid hydroxylase	Mao et al., 2012
*OsGL1-6*	*O. sativa*	Aldehyde decarbonylase	Zhou et al., 2013
*DWA1*	*O. sativa*	Fatty acyl-AMP ligase	Zhu and Xiong, 2013
*EsWAX1*	*E. salsugineum*	MYB transcription factors	Zhu et al., 2014
*MYB94*	*A. thaliana*	MYB transcription factors	Lee and Suh, 2015b
*CsWAX2*	*C. sativus*	Aldehyde decarbonylase	Wang et al., 2015
*TaFAR2/3/4*	*T. aestivum*	Fatty acyl-coenzyme A reductase	Wang et al., 2016

The biosynthesis and transport of cuticular wax have been characterized in *Arabidopsis* wax-deficient mutants. Cuticular wax loads increased approximately twofold under drought in *Arabidopsis*, which is achieved by regulating genes related to wax biosynthesis (Kosma et al., 2009). *AtCER1* controls the biosynthesis of alkane and is associated with drought stress responses (Bourdenx et al., 2011). *AtCER1* encodes a novel protein involved in wax biosynthesis for long chain aldehydes into alkanes (Aarts et al., 1995). *AtCER1* is induced by osmotic stress and is specifically expressed in the epidermis of aerial organs. The overexpression of *AtCER1* significantly increases the production of iso-branched and odd-carbon-numbered alkanes. Importantly, *AtCER1*-overexpressing plants showed increased drought tolerance (Bourdenx et al., 2011). It has been found that *AtCER6* is involved in the VLCFA precursor biosynthesis for wax production exclusively in the epidermal cells in all examined tissues of shoots and is highly transcribed throughout development. Environmental factors were shown to up-regulate *AtCER6* to stimulate wax accumulation (Hooker et al., 2002). In addition, ABA treatment resulted in threefold greater expression of *AtCER6* in shoot, suggesting that drought hormone ABA is related to the transcription of wax synthesis gene *AtCER6* (Hooker et al., 2002). In *Atwax2* mutant, the proportional deficiencies in alkanes, secondary alcohols, aldehydes, and ketones were found and the total amount of stem and leaf wax was reduced by 78% (Chen et al., 2003). It was found that *AtWAX2* is required for both cuticular wax and cutin deposition. The AtWAX2 protein exhibits 32% similarity to AtCER1 with certain homologous regions to sterol desaturases and short-chain dehydrogenases/reductases (Chen et al., 2003). In cucumber, the expression of *CsWAX2* can be induced by drought, ABA, low temperature, and salinity. In comparison to the WT, the *CsWAX2* transgenic cucumber plants showed significant improvement in resistance to drought and pathogens. These results suggested that *CsWAX2* plays an important role in the biosynthesis of wax, influencing the response to biotic and abiotic stresses in cucumber (Wang et al., 2015). Karaba (2007) transferred *AtCER1*, *AtCER6* and *AtWAX2*, which are involved in the pathways of wax biosynthesis, into tomato and the transformed tomato lines exhibited enhanced epicuticular wax predominantly consisted of alkanes. Compared with WT, transgenic tomato lines showed reduced water loss and enhanced drought tolerance. These tomato lines also showed a significant increase in WUE and no reduction in biomass under drought stress (Karaba, 2007).

Drought stress significantly influences the biosynthesis and composition of cuticular wax in crops, affecting crop yield. Some genes involved in cuticular wax accumulation with drought stress have been reported in crops. In rice, *OsWSL1*, one of the *3-ketoacyl-CoA synthase* (*KCS*) genes, catalyzes the formation of C_20_ to C_24_ VLCFA precursors of leaf waxes. The *Oswsl1* mutant showed a pleiotropic phenotype with decreased growth, sparse wax crystals and drought sensitivity to, suggesting that *OsWSL1* may be relevant to drought tolerance (Yu et al., 2008). Recently, Zhou et al. (2013) identified a rice fatty aldehyde decarbonylase gene *OsGL1-6*, which is expressed in epidermal cells as well as in vascular bundles and is required for formation of wax on leaf blades. Decreased expression of *OsGL1-6* was linked to obviously decrease of total wax and increased drought sensitivity (Zhou et al., 2013). Compared to the WT, *Osgl1-2* and *Osgl1-1/Oswsl2* mutants have been identified to exhibit increased sensitivity to drought stress and reduced wax load (Islam et al., 2009; Qin et al., 2011; Mao et al., 2012). Zhu and Xiong (2013) identified the *Drought-induced Wax Accumulation1* (*OsDWA1*) gene in rice and found OsDWA1 contains an AMP-binding domain for the long chain fatty acids into acyl-CoAs. It was found that *OsDWA1*-overexpressing plants show enhanced VLCFA production and improves drought tolerance. *OsDWA1* expression was also increased by up to 4.9-fold in ABA treatment. In tomato, it was found that water loss is increased in the fruit of the *Lecer6* mutant, demonstrating that the composition and distribution of cuticular wax are crucial factors of the fruit transpiration barrier (Leide et al., 2007). These findings may have significant implications for improving drought resistance in crops.

Drought stress induces the synthesis and transport of ABA, causing stomatal closure and inducing expression of many abiotic stress-related genes. It was found that ABA induces upregulation of 10 out of 25 cuticle-associated genes, including *acetyl-CoA carboxylase 1* (*ACC1*), *CER1, CER2, CER5, CER6, CER60, CYP86A2 (cytochrome P450, family 86, subfamily A, polypeptide 2), KCS1*, *long-chain acyl-CoA synthetase 2 (LACS2), WAX2/YRE* (Kosma and Jenks, 2007). However, limited number of these genes has been verified experimentally. Wang et al. (2016) identified and characterized three fatty acyl-coenzyme A reductase-encoding wheat genes: *TaFAR2, TaFAR3*, and *TaFAR4*. Transgenic and transcriptional analysis showed that *TaFARs* encode active alcohol-forming FARs, affecting the biosynthesis of wax and the response to drought and ABA in wheat (Wang et al., 2016). Along with the isolation and functional analysis of wax-related genes, molecular mechanisms of cuticular wax and its relationship with ABA is likely to be elucidated in the near future.

## Molecular Regulation of Transcription Factors on Cuticular Wax for Plant Drought Tolerance

A few TFs (**Table 1**) have been implicated for cuticular wax biosynthesis and accumulation under drought stress (Aharoni et al., 2004; Zhang et al., 2005, 2007; Cominelli et al., 2008; Seo et al., 2011). *AtSHNs* encode proteins of APETALA 2/Ethylene Response Element Binding Protein (AP2/EREBP) TFs. Overexpressing *AtSHN* altered epidermal properties and increased epicuticular wax in *Arabidopsis*. Interestingly, *AtSHN* overexpressors-induced important drought tolerance probably links with the reduced stomatal density, and *AtSHN2* and *AtSHN3* were also reported to have similar functions to *AtWIN1/AtSHN1* (Aharoni et al., 2004; Broun et al., 2004). It has been found that *Arabidopsis* plants overexpressing AP2/EREBP TFs *MtWXP1* and *MtWXP2* from *Medicago truncatula* affect accumulation of cuticular wax and show enhanced drought resistance (Zhang et al., 2005, 2007). In *Arabidopsis*, overexpression of *AtWIN1* has been reported to confer drought tolerance (Aharoni et al., 2004). In the following work, it was found that *AtWIN1/AtSHN1* directly controls the expression of the *AtLACS2 (long-chain acyl-CoA synthetase 2), AtGPAT4 (glycerol-3-phosphate acyltransferase 4), AtCYP86A4, AtCYP86A7*, and HTH-like genes only regulates the deposition of cuticular wax indirectly (Kannangara et al., 2007).

MYB TFs are featured by the unique MYB domain participating DNA binding. Recently, a number of *MYBs* have been identified for the participation in regulating drought stress tolerance in plants (Baldoni et al., 2015). Different *Arabidopsis* R2R3-MYB TFs have been reported to regulate cuticular components biosynthesis. However, only two members of the MYB TF family, *At*MYB94 and *At*MYB96, are recognized as regulatory components for the biosynthesis of wax in drought (Cominelli et al., 2008; Seo et al., 2011; Lee and Suh, 2015b). It was found that *AtMYB96* regulates the whole wax metabolism, and the cuticular wax biosynthetic genes *AtKCS1, AtKCS2, AtKCS6, AtKCR1*, and *AtCER3* were identified as direct targets of MYB96 (Seo et al., 2009, 2011; Lee and Suh, 2013). Seo et al. (2009) reported that *AtMYB96* is up-regulated in ABA treatments and that expression levels of MYB96 are positively correlated with the resistance to drought in plants. Key genes *KCSs, 3-ketoacyl-CoA reductases 1 (KCR1), CER10, 3-hydroxyacyl-CoA dehydratase2 (PAS2), CER4, WAX ESTER SYNTHASE/ACYL-COA: DIACYLGLYCEROL ACYLTRANSFERASE1 (WSD1), CER3, CER1*, and *MID-CHAIN ALKANE HYDROXYLASE 1(MAH1)* were up-regulated in the *AtMYB96* overexpressing lines (Seo et al., 2011). Overexpressing *AtMYB96* in *Atmyb96* mutant led to an increase in all wax compounds while the wax load decreased. The results suggested that ABA induction of wax metabolism is dependent on *At*MYB96 (Seo et al., 2011). In addition, the ectopic expression of *AtMYB96* in *Camelina sativa*, also showed a significant up-regulation of wax biosynthesis, an enhanced accumulation of wax load, and increased drought tolerance of transgenic plants (Lee et al., 2014). Recently, it was reported that *AtLTP3*, which encodes a lipid-transfer protein, is strongly regulated by *AtMYB96* via the direct binding to the *AtLTP3* promoter and acts as a target of *AtMYB96* to participate in plant tolerance to drought (Guo et al., 2013). In *Eutrema salsugineum*, the *EsWAX1* was identified as a TF similar to *AtMYB96*. The transcript of *EsWAX1* was significantly activated in response to drought and ABA. In *Arabidopsis*, *EsWAX1* overexpressor enhanced the expression of several wax-associated genes like *AtCER1, AtKCS1*, and *AtKCR1* and accumulated cuticular wax. These results suggested that *EsWAX1* may play an essential role in ABA-mediated drought stress response (Zhu et al., 2014). Also, *AtMYB94* was found to initiate the biosynthesis of cuticular wax via the up-regulation of *AtWSD1, AtKCS2/AtDAISY, AtCER2, AtFAR3*, and *AtECR*. In the leaves of *AtMYB94* overexpression, it was observed an increase in the cuticular wax accumulation to reduce cuticular transpiration rate under drought stress. These demonstrate that certain MYB TFs act as transcriptional activators for biosynthesis and accumulation of wax in response to drought stress.

WRKY proteins are a large family of transcriptional regulators for the modulation of various developmental and stress responses in plants. Three groups of WRKYs were reported based on the number of WRKY domains and the zinc-finger motif pattern (Phukan et al., 2016). For instance, *OsWRKY89* may play an important role in response to drought in rice. It was reported that leaf surface wax deposition increases in the *OsWRKY89* overexpression lines and wax loading decreases in the *OsWRKY89* RNAi lines. However, no cuticular wax biosynthesis genes have been found so far to be regulated by *OsWRKY89* (Wang et al., 2007). Future studies should focus on the discovery of potential WRKY TFs that may regulate cuticular wax biosynthesis genes.

## Molecular Evolution of Cuticular Wax for Plant Drought Tolerance

**FIGURE 2:**
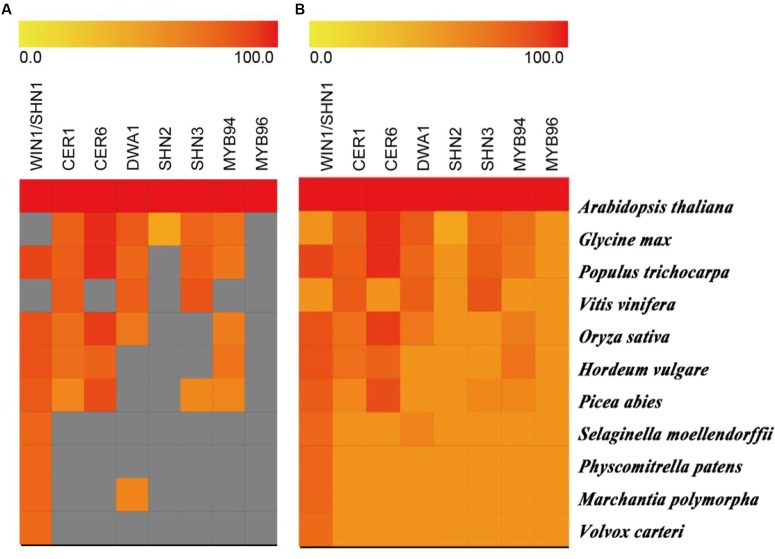
**Similarity heat map of key cuticular wax-related genes involved in drought tolerance in different species**. Genesis software was used to estimate the similarity among selected protein sequences of different plant and algal species. Candidate protein sequences were selected by BLASTP searches which satisfied E-value < 10^-10^ and query coverage > 50% **(A)** and an E-value < 10^-5^ **(B)**. Colored squares indicate protein sequence similarity from zero (yellow) to 100 (red). Gray squares indicate proteins that are satisfied with the selection criteria.

There are many cuticular wax-related genes regulating drought tolerance in flowering plants. Are these genes conserved in all plant lineages? Is there a stepwise evolution of these genes in plants? When plants began to colonize the terrestrial habitat, their living environment significantly changed with large fluctuation of temperature, radiation and water availability, as compared to aquatic environments (Cai et al., 2017; Chen et al., 2017; and reference within). Therefore, we hypothesized that there is an adaptive and stepwise evolution of cuticular wax related genes in land plants. Here, we conducted a comparative genomics analysis of the cuticular wax-related key genes involved in drought tolerance to provide some insights (**Figure 2**). Based on the strict selection criteria (E-value < 10^-10^ and query coverage > 50%), we found that algae, liverwort, moss, and lycophyte may not have evolved many of the cuticular wax related proteins with the exception of TF WIN1/SHN1 (**Figure 2A**), although orthologous proteins are found in all tested plant species with the less stringent selection criteria (E-value < 10^-5^). The orthologous proteins of CER1, CER6, and DWA1 for cuticular wax biosynthesis have been identified in all the selected gymnosperm and angiosperm species. Similarly, the TFs related to cuticular wax for plant drought tolerance are not found in algae, liverwort, and moss (**Figure 2**). The results suggested that higher plants may have evolved more proteins for biosynthesis and transport of cuticular wax for the adaption to dry environments.

We made the follow speculations about the evolution of key cuticular wax proteins based on this review. Higher plants have cuticles to prevent water loss and adapt drought conditions. Although there is no need to protect against water loss in green algae lives in aquatic environments, some green algae have been found living on land as filaments or single-cell layer thick sheets forming ground-hugging mats at wet ground to reduce the chance of desiccation. These algae probably represent first species during the evolution of cuticular wax. Bryophytes are usually restricted to moist environments, but they require higher level of complexity in cuticular wax for terrestrially living in contrast to algae. When the evolution of plant researches to gymnosperm and angiosperm species, biosynthesis, transport, and molecular regulation of cuticular wax become an essential components and adaption to drought conditions, which is reflected by the increasingly complex and number of genes and proteins related to cuticular wax (**Figure 2** and Supplementary Table S1). These again validate from an evolutionary point of view that cuticular wax is amongst the most critical evolutionary components of plant drought tolerance. However, further studies are necessary to conduct functional analysis of these proteins and to identify more candidate genes involved in cuticular wax in different species along the plant evolutionary lineages.

## Concluding Remarks and Future Perspectives

Drought stress seriously affects the growth and production of plants including crops, and the breeding of crops with tolerance to drought stress has become an urgent unresolved problem. There is a growing understanding of the relationship between the drought stress and the morphology and composition of cuticular wax, which has laid the foundation for further studies on the functions and mechanisms of wax under drought stress. However, the relationship between the cuticular wax content, composition, and morphology of the structure and drought tolerance still lacks of in-depth study. To date, there are a number of cuticular wax-related genes have been identified in close association with drought stress. However, the detailed function and regulation of these genes need to be studied further, especially on the key genes of wax metabolism, including their involvement in wax metabolic regulation in the specific role and regulation. In particular, understanding how ABA participates in cuticular wax metabolic regulation will shed some light for future research. In addition, functional proteins relevant to cuticular wax transport and TFs regulating the expression of key genes related to network of cuticular wax synthesis and secretion under drought conditions remain to be revealed. Therefore, understanding the roles of plant cuticular wax in abiotic stress tolerance and the effect of molecular mechanisms of wax synthesis will have important knowledge advancement, resulting in the addition of huge practical values to the agricultural sectors. Knowledge of biosynthetic machinery, transport, and utilization of cuticular wax in diverse plant species will benefit the development of crop cultivars with improved drought tolerance for increasing yield and quality contributing to sustainable agriculture.

## Author Contributions

DX and Z-HC: conceived the research work. XL and GC: conducted bioinformatics analysis. DX, XZ, XL, GC, and Z-HC: prepared the figures and tables. DX and Z-HC: wrote the paper.

## Conflict of Interest Statement

The authors declare that the research was conducted in the absence of any commercial or financial relationships that could be construed as a potential conflict of interest.

## References

[B1] Aarts M. G. Keijzer C. J. Stiekema W. J. Pereira A. (1995). Molecular characterization of the CER1 gene of arabidopsis involved in epicuticular wax biosynthesis and pollen fertility. *Plant Cell* 7 2115–2127. 8718622 10.1105/tpc.7.12.2115 PMC161066

[B2] Aharoni A. Dixit S. Jetter R. Thoenes E. van Arkel G. Pereira A. (2004). The SHINE clade of AP2 domain transcription factors activates wax biosynthesis, alters cuticle properties, and confers drought tolerance when overexpressed in Arabidopsis. *Plant Cell* 16 2463–2480. 10.1105/tpc.104.022897 15319479 PMC520946

[B3] Albert Z. Ivanics B. Molnár A. Miskó A. Tóth M. Papp I. (2013). Candidate genes of cuticle formation show characteristic expression in the fruit skin of apple. *Plant Growth Regul.* 70 71–78. 10.1007/s10725-012-9779-y

[B4] Baldoni E. Genga A. Cominelli E. (2015). Plant MYB transcription factors: their role in drought response mechanisms. *Int. J. Mol. Sci.* 16 15811–15851. 10.3390/ijms160715811 26184177 PMC4519927

[B5] Bernard A. Joubès J. (2013). Arabidopsis cuticular waxes: advances in synthesis, export and regulation. *Prog. Lipid Res.* 52 110–129. 10.1016/j.plipres.2012.10.002 23103356

[B6] Bird D. Beisson F. Brigham A. Shin J. Greer S. Jetter R. (2007). Characterization of Arabidopsis ABCG11/WBC11, an ATP binding cassette (ABC) transporter that is required for cuticular lipid secretion. *Plant J.* 52 485–498. 10.1111/j.1365-313X.2007.03252.x 17727615

[B7] Bourdenx B. Bernard A. Domergue F. Pascal S. Leger A. Roby D. (2011). Overexpression of Arabidopsis *ECERIFERUM1* promotes wax very-long-chain alkane biosynthesis and influences plant response to biotic and abiotic stresses. *Plant Physiol.* 156 29–45. 10.1104/pp.111.172320 21386033 PMC3091054

[B8] Broun P. Poindexter P. Osborne E. Jiang C. Z. Riechmann J. L. (2004). WIN1, a transcriptional activator of epidermal wax accumulation in *Arabidopsis*. *Proc. Natl. Acad. Sci. U.S.A.* 101 4706–4711. 10.1073/pnas.0305574101 15070782 PMC384811

[B9] Buda G. J. Barnes W. J. Fich E. A. Park S. Yeats T. H. Zhao L. (2013). An ATP binding cassette transporter is required for cuticular wax deposition and desiccation tolerance in the moss *Physcomitrella patens*. *Plant Cell* 25 4000–4013. 10.1105/tpc.113.117648 24163310 PMC3877811

[B10] Burow G. B. Franks C. D. Xin Z. (2008). Genetic and physiological analysis of an irradiated bloomless mutant (epicuticular wax mutant) of *Sorghum*. *Crop Sci.* 48 41–48. 10.2135/cropsci2007.02.0119

[B11] Buschhaus C. Jetter R. (2011). Composition differences between epicuticular and intracuticular wax substructures: how do plants seal their epidermal surfaces? *J. Exp. Bot.* 62 841–853. 10.1093/jxb/erq366 21193581

[B12] Cai S. Chen G. Wang Y. Huang Y. Marchant B. Wang Y. (2017). Evolutionary conservation of ABA signaling for stomatal closure in ferns. *Plant Physiol.* 10.1104/pp.16.01848 [Epub ahead of print]. PMC5462018 28232585

[B13] Caldicott A. B. Eglinton G. (1976). Cutin acids from bryophytes: an ω-1 hydroxy alkanoic acid in two liverwort species. *Phytochemistry* 15 1139–1143. 10.1016/0031-9422(76)85118-7

[B14] Cameron K. D. Teece M. A. Smart L. B. (2006). Increased accumulation of cuticular wax and expression of lipid transfer protein in response to periodic drying events in leaves of tree tobacco. *Plant Physiol.* 140 176–183. 10.1104/pp.105.069724 16361524 PMC1326042

[B15] Chen X. Goodwin S. M. Boroff V. L. Liu X. Jenks M. A. (2003). Cloning and characterization of the *WAX2* gene of Arabidopsis involved in cuticle membrane and wax production. *Plant Cell* 15 1170–1185. 10.1105/tpc.010926 12724542 PMC153724

[B16] Chen Z. Newman I. Zhou M. Mendham N. Zhang G. Shabala S. (2005). Screening plants for salt tolerance by measuring K+ flux: a case study for barley. *Plant Cell Environ.* 28 1230–1246. 10.1111/j.1365-3040.2005.01364.x

[B17] Chen Z. H. Chen G. Dai F. Wang Y. Hills A. Ruan Y. L. (2017). Molecular evolution of grass stomata. *Trends Plant Sci.* 22 124–139. 10.1016/j.tplants.2016.09.005 27776931

[B18] Comas L. H. Becker S. R. Cruz V. M. Byrne P. F. Dierig D. A. (2013). Root traits contributing to plant productivity under drought. *Front. Plant Sci.* 4:442 10.3389/fpls.2013.00442 PMC3817922 24204374

[B19] Cominelli E. Sala T. Calvi D. Gusmaroli G. Tonelli C. (2008). Over-expression of the Arabidopsis *AtMYB41* gene alters cell expansion and leaf surface permeability. *Plant J.* 53 53–64. 10.1111/j.1365-313X.2007.03310.x 17971045

[B20] Cook M. E. Graham L. E. (1998). Structural similarities between surface layers of selected charophycean algae and bryophytes and the cuticles of vascular plants. *Int. J. Plant Sci.* 159 780–787. 10.1086/297597

[B21] Dai F. Chen Z. H. Wang X. Li Z. Jin G. Wu D. (2014). Transcriptome profiling reveals mosaic genomic origins of modern cultivated barley. *Proc. Natl. Acad. Sci. U.S.A.* 111 13403–13408. 10.1073/pnas.1414335111 25197090 PMC4169977

[B22] DeBono A. Yeats T. H. Rose J. K. Bird D. Jetter R. Kunst L. (2009). *Arabidopsis* LTPG is a glycosylphosphatidylinositol-anchored lipid transfer protein required for export of lipids to the plant surface. *Plant Cell* 21 1230–1238. 10.1105/tpc.108.064451 19366900 PMC2685631

[B23] Domínguez E. Heredia-Guerrero J. A. Heredia A. (2011). The biophysical design of plant cuticles: an overview. *New Phytol.* 189 938–949. 21374891 10.1111/j.1469-8137.2010.03553.x

[B24] Edwards D. Abbott G. D. Raven J. A. (1996). “Cuticles of early land plants: a palaeoecophysiological evaluation,” in *Plant Cuticles: An Integrated Functional Approach*, ed. Kerstiens G. (Oxford: Bios Scientific Publishers), 1–31.

[B25] Fang Y. Xiong L. (2015). General mechanisms of drought response and their application in drought resistance improvement in plants. *Cell. Mol. Life Sci.* 72 673–689. 10.1007/s00018-014-1767-0 25336153 PMC11113132

[B26] Farooq M. Wahid A. Kobayashi N. Fujita D. Basra S. M. A. (2009). Plant drought stress: effects, mechanisms and management. *Agron. Sustain. Dev.* 29 185–212.

[B27] Febrero A. Fernández S. Molina-Cano J. L. Araus J. L. (1998). Yield, carbon isotope discrimination, canopy reflectance and cuticular conductance of barley isolines of differing glaucousness. *J. Exp. Bot.* 49 1575–1581. 10.1093/jxb/49.326.1575

[B28] Fernández V. Guzmán-Delgado P. Graca J. Santos S. Gil L. (2016). Cuticle structure in relation to chemical composition: re-assessing the prevailing model. *Front. Plant Sci.* 7:427 10.3389/fpls.2016.00427 PMC4814898 27066059

[B29] Fich E. A. Segerson N. A. Rose J. K. (2016). The plant polyester cutin: biosynthesis, structure, and biological roles. *Annu. Rev. Plant Biol.* 67 207–233. 10.1146/annurev-arplant-043015-111929 26865339

[B30] Fukuda S. Satoh A. Kasahara H. Matsuyama H. Takeuchi Y. (2008). Effects of ultraviolet-B irradiation on the cuticular wax of cucumber (*Cucumis sativus*) cotyledons. *J. Plant Res.* 121 179–189. 10.1007/s10265-007-0143-7 18217194

[B31] Geyer U. Schönherr J. (1990). The effect of the environment on the permeability and composition of *Citrus* leaf cuticles:I. Water permeability of isolated cuticular membranes. *Planta* 180 147–153. 10.1007/bf00193989 24201938

[B32] González A. Ayerbe L. (2010). Effect of terminal water stress on leaf epicuticular wax load, residual transpiration and grain yield in barley. *Euphytica* 172 341–349. 10.1007/s10681-009-0027-0

[B33] Graham L. E. (1993). *Origin of Land Plants.* New York, NY: Wiley and Sons.

[B34] Guo J. Xu W. Yu X. Shen H. Li H. Cheng D. (2016). Cuticular wax accumulation is associated with drought tolerance in wheat near-isogenic lines. *Front. Plant Sci.* 7:1809 10.3389/fpls.2016.01809 PMC5129171 27965701

[B35] Guo L. Yang H. Zhang X. Yang S. (2013). *Lipid transfer protein 3* as a target of MYB96 mediates freezing and drought stress in *Arabidopsis*. *J. Exp. Bot.* 64 1755–1767. 10.1093/jxb/ert040 23404903 PMC3617838

[B36] Holloway P. J. (1982). “The chemical constitution of plant cutins,” in *The Plant Cuticle*, eds Cutler D. F. Alvin K. L. Price C. E. (London: Academic Press), 45–85.

[B37] Honsbein A. Sokolovski S. Grefen C. Campanoni P. Pratelli R. Paneque M. (2009). A tripartite SNARE-K+ channel complex mediates in channel-dependent K+ nutrition in *Arabidopsis*. *Plant Cell* 21 2859–2877. 10.1105/tpc.109.066118 19794113 PMC2768940

[B38] Hooker T. S. Millar A. A. Kunst L. (2002). Significance of the expression of the *CER6* condensing enzyme for cuticular wax production in Arabidopsis. *Plant Physiol.* 129 1568–1580. 10.1104/pp.003707 12177469 PMC166744

[B39] Hunneman D. H. Eglinton G. (1972). The constituent acids of gymnosperm cutins. *Phytochemistry* 11 1989–2001. 10.1016/S0031-9422(00)90163-8

[B40] Islam M. A. Du H. Ning J. Ye H. Xiong L. (2009). Characterization of Glossy1-homologous genes in rice involved in leaf wax accumulation and drought resistance. *Plant Mol. Biol.* 70 443–456. 10.1007/s11103-009-9483-0 19322663

[B41] Jansen M. A. K. Gaba V. Greenberg B. M. (1998). Higher plants and UV-B radiation: balancing damage, repair and acclimation. *Trends Plant Sci.* 3 243–243. 10.1016/S1360-1385(98)01215-1

[B42] Javelle M. Vernoud V. Rogowsky P. M. Ingram G. C. (2011). Epidermis: the formation and functions of a fundamental plant tissue. *New Phytol.* 189 17–39. 10.1111/j.1469-8137.2010.03514.x 21054411

[B43] Jeffree C. E. (2006). “The fine structure of the plant cuticle,” in *Biology of the Plant Cuticle, Annual Plant Reviews* Vol. 23 eds Riederer M. M C.üller (Oxford: Blackwell Publishing), 11–125.

[B44] Jenks M. A. Andersen L. Teusink R. S. Williams M. H. (2001). Leaf cuticular waxes of potted rose cultivars as affected by plant development, drought and paclobutrazol treatments. *Physiol. Plant.* 112 62–70. 10.1034/j.1399-3054.2001.1120109.x 11319016

[B45] Jetter R. Kunst L. Samuels A. L. (2006). “Composition of plant cuticular waxes,” in *Biology of the Plant Cuticle, Annual Plant Reviews* Vol. 23 eds Riederer M. M C.üller (Oxford: Blackwell Publishing), 145–181.

[B46] Jiang L. Wang Y. Bjorn L. O. Li S. S. (2009). Arabidopsis *RADICAL-INDUCED CELL DEATH1* is involved in UV-B signaling. *Photochem. Photobiol. Sci.* 8 838–846. 10.1039/b901187k 19492112

[B47] Johnson D. A. Richards R. A. Turner N. C. (1983). Yield, water relations, gas exchange, and surface reflectances of near-isogenic wheat lines differing in glaucousness. *Crop Sci.* 23 318–325. 10.2135/cropsci1983.0011183X002300020033x

[B48] Jordan W. R. Shouse P. J. Blum A. Miller F. R. Monk R. L. (1984). Environmental physiology of sorghum. II. epicuticular wax load and cuticular transpiration. *Crop Sci.* 24 1168–1173. 10.2135/cropsci1984.0011183X002400060038x

[B49] Joshi R. Wani S. H. Singh B. Bohra A. Dar Z. A. Lone A. A. (2016). Transcription factors and plants response to drought stress: current understanding and future directions. *Front. Plant Sci.* 7:1029 10.3389/fpls.2016.01029 PMC4943945 27471513

[B50] Kamigaki A. Kondo M. Mano S. Hayashi M. Nishimura M. (2009). Suppression of peroxisome biogenesis factor 10 reduces cuticular wax accumulation by disrupting the ER network in *Arabidopsis thaliana*. *Plant Cell Physiol.* 50 2034–2046. 10.1093/pcp/pcp152 19892830

[B51] Kannangara R. Branigan C. Liu Y. Penfield T. Rao V. Mouille G. (2007). The transcription factor WIN1/SHN1 regulates cutin biosynthesis in *Arabidopsis thaliana*. *Plant Cell* 19 1278–1294. 10.1105/tpc.106.047076 17449808 PMC1913754

[B52] Karaba A. (2007). *Improvement of Water Use Efficiency in Rice and Tomato Using Arabidopsis Wax Biosynthetic Genes and Transcription Factors.* Ph.D. thesis, Wageningen University, Wageningen.

[B53] Kenrick P. Crane P. R. (1997). The origin and early evolution of plants on land. *Nature* 389 33–39. 10.1038/37918

[B54] Kim H. Lee S. B. Kim H. J. Min M. K. Hwang I. Suh M. C. (2012). Characterization of glycosylphosphatidylinositol-anchored lipid transfer protein 2 (LTPG2) and overlapping function between LTPG/LTPG1 and LTPG2 in cuticular wax export or accumulation in *Arabidopsis thaliana*. *Plant Cell Physiol.* 53 1391–1403. 10.1093/pcp/pcs083 22891199

[B55] Knight T. G. Wallwork M. A. B. Sedgley M. (2004). Leaf epicuticular wax and cuticle ultrastructure of foureucalyptusspecies and their hybrids. *Int. J. Plant Sci.* 165 27–36. 10.1086/380744

[B56] Kolattukudy P. E. (1996). “Biosynthetic pathways of cutin and waxes, their sensitivity to environmental stresses,” in *Plant Cuticles: An Integrated Functional Approach*, ed. Kerstiens G. (Oxford: Bios Scientific Publishers), 83–108.

[B57] Kosma D. K. Bourdenx B. Bernard A. Parsons E. P. Lu S. Joubes J. (2009). The impact of water deficiency on leaf cuticle lipids of Arabidopsis. *Plant Physiol.* 151 1918–1929. 10.1104/pp.109.141911 19819982 PMC2785987

[B58] Kosma D. K. Jenks M. A. (2007). “Eco-physiological and molecular-genetic determinants of plant cuticle function in drought and salt stress tolerance,” in *Advances in Molecular Breeding Toward drought and Salt Tolerant Crops*, eds Jenks M. A. Hasegawa P. M. Jain S. M. (Dordrecht: Springer Publishing), 91–120.

[B59] Kunst L. Samuels A. L. (2003). Biosynthesis and secretion of plant cuticular wax. *Prog. Lipid Res.* 42 51–80. 12467640 10.1016/s0163-7827(02)00045-0

[B60] Kunst L. Samuels L. (2009). Plant cuticles shine: advances in wax biosynthesis and export. *Curr. Opin. Plant Biol.* 12 721–727. 10.1016/j.pbi.2009.09.009 19864175

[B61] Lee S. B. Jung S. J. Go Y. S. Kim H. U. Kim J. K. Cho H. J. (2009). Two Arabidopsis 3-ketoacyl CoA synthase genes, *KCS20* and *KCS2/DAISY*, are functionally redundant in cuticular wax and root suberin biosynthesis, but differentially controlled by osmotic stress. *Plant J.* 60 462–475. 10.1111/j.1365-313X.2009.03973.x 19619160

[B62] Lee S. B. Kim H. Kim R. J. Suh M. C. (2014). Overexpression of Arabidopsis *MYB96* confers drought resistance in *Camelina sativa* via cuticular wax accumulation. *Plant Cell Rep.* 33 1535–1546. 10.1007/s00299-014-1636-1 24880908

[B63] Lee S. B. Suh M. C. (2013). Recent advances in cuticular wax biosynthesis and its regulation in *Arabidopsis*. *Mol. Plant* 6 246–249. 10.1093/mp/sss159 23253604

[B64] Lee S. B. Suh M. C. (2015a). Advances in the understanding of cuticular waxes in *Arabidopsis thaliana* and crop species. *Plant Cell Rep.* 34 557–572. 10.1007/s00299-015-1772-2 25693495

[B65] Lee S. B. Suh M. C. (2015b). Cuticular wax biosynthesis is up-regulated by the MYB94 transcription factor in Arabidopsis. *Plant Cell Physiol.* 56 48–60. 10.1093/pcp/pcu142 25305760

[B66] Leide J. Hildebrandt U. Reussing K. Riederer M. Vogg G. (2007). The developmental pattern of tomato fruit wax accumulation and its impact on cuticular transpiration barrier properties: effects of a deficiency in b-ketoacyl-coenzyme a synthase (LeCER6). *Plant Physiol.* 144 1667–1679. 10.1104/pp.107.099481 17468214 PMC1914139

[B67] Liu X. Mak M. Babla M. Wang F. Chen G. Veljanoski F. (2014). Linking stomatal traits and expression of slow anion channel genes *HvSLAH1* and *HvSLAC1* with grain yield for increasing salinity tolerance in barley. *Front. Plant Sci.* 5:634 10.3389/fpls.2014.00634 PMC4243495 25505473

[B68] Majada J. Sierra M. Sanchez-Tames R. (2001). Air exchange rate affects the in vitro developed leaf cuticle of carnation. *Sci. Hortic.* 87 121–130. 10.1016/S0304-4238(00)00162-X

[B69] Mak M. Babla M. Xu S.-C. O’Carrigan A. Liu X.-H. Gong Y.-M. (2014). Leaf mesophyll K+, H+ and Ca2+ fluxes are involved in drought-induced decrease in photosynthesis and stomatal closure in soybean. *Environ. Exp. Bot.* 98 1–12. 10.1016/j.envexpbot.2013.10.003

[B70] Mao B. Cheng Z. Lei C. Xu F. Gao S. Ren Y. (2012). Wax crystal-sparse leaf2, a rice homologue of WAX2/GL1, is involved in synthesis of leaf cuticular wax. *Planta* 235 39–52. 10.1007/s00425-011-1481-1 21809091

[B71] Monneveux P. Reynolds M. P. González-Santoyo H. Peña R. J. Mayr L. Zapata F. (2004). Relationships between grain yield, flag leaf morphology, carbon isotope discrimination and ash content in irrigated wheat. *J. Agron. Crop Sci.* 190 395–401. 10.1111/j.1439-037X.2004.00116.x

[B72] Moreshet S. (1970). Effect of environmental factors on cuticular transpiration resistance. *Plant Physiol.* 46 815–818. 16657551 10.1104/pp.46.6.815 PMC396688

[B73] Nawrath C. Schreiber L. Franke R. B. Geldner N. Reina-Pinto J. J. Kunst L. (2013). Apoplastic diffusion barriers in Arabidopsis. *Arabidopsis Book* 11:e0167 10.1199/tab.0167 PMC3894908 24465172

[B74] Ni Y. Xia R. E. Li J. N. (2014). Changes of epicuticular wax induced by enhanced UV-B radiation impact on gas exchange in *Brassica napus*. *Acta Physiol. Plant.* 36 2481–2490. 10.1007/s11738-014-1621-x

[B75] O’Carrigan A. Hinde E. Lu N. Xu X. Q. Duan H. Huang G. (2014). Effects of light irradiance on stomatal regulation and growth of tomato. *Environ. Exp. Bot.* 98 65–73. 10.1016/j.envexpbot.2013.10.007

[B76] Panikashvili D. Savaldi-Goldstein S. Mandel T. Yifhar T. Franke R. B. Höfer R. (2007). The Arabidopsis *DESPERADO/AtWBC11* transporter is required for cutin and wax secretion. *Plant Physiol.* 145 1345–1360. 10.1104/pp.107.105676 17951461 PMC2151707

[B77] Park J. J. Jin P. Yoon J. Yang J. I. Jeong H. J. Ranathunge K. (2010). Mutation in *Wilted Dwarf and Lethal 1 (WDL1)* causes abnormal cuticle formation and rapid water loss in rice. *Plant Mol. Biol.* 74 91–103. 10.1007/s11103-010-9656-x 20593223

[B78] Pennisi E. (2008). Plant genetics. The blue revolution, drop by drop, gene by gene. *Science* 320 171–173. 10.1126/science.320.5873.171 18403686

[B79] Phukan U. J. Jeena G. S. Shukla R. K. (2016). WRKY transcription factors: molecular regulation and stress responses in plants. *Front. Plant Sci.* 7:760 10.3389/fpls.2016.00760 PMC4891567 27375634

[B80] Pighin J. A. Zheng H. Balakshin L. J. Goodman I. P. Western T. L. Jetter R. (2004). Plant cuticular lipid export requires an ABC transporter. *Science* 306 702–704. 10.1126/science.1102331 15499022

[B81] Pornsiriwong W. Estavillo G. M. Chan K. X. Tee E. E. Ganguly D. Crisp P. A. (2017). A chloroplast retrograde signal, 3’-phosphoadenosine 5’-phosphate, acts as a secondary messenger in abscisic acid signaling in stomatal closure and germination. *ELife* 6:e23361 10.7554/eLife.23361 PMC5406205 28323614

[B82] Qin B. X. Tang D. Huang J. Li M. Wu X. R. Lu L. L. (2011). Rice *OsGL1*-1 is involved in leaf cuticular wax and cuticle membrane. *Mol. Plant* 4 985–995. 10.1093/mp/ssr028 21511810

[B83] Richards R. A. Rawson H. M. Johnson D. A. (1986). Glaucousness in wheat: its development and effect on water-use efficiency, gas exchange and photosynthetic tissue temperatures. *Funct. Plant Biol.* 13 465–473. 10.1071/PP9860465

[B84] Riederer M. Schreiber L. (2001). Protecting against water loss: analysis of the barrier properties of plant cuticles. *J. Exp. Bot.* 52 2023–2032. 11559738 10.1093/jexbot/52.363.2023

[B85] Samuels L. Kunst L. Jetter R. (2008). Sealing plant surfaces: cuticular wax formation by epidermal cells. *Annu. Rev. Plant Biol.* 59 683–707. 10.1146/annurev.arplant.59.103006.093219 18251711

[B86] Sánchez F. J. Manzanares M. de Andrés E. F. Tenorio J. L. Ayerbe L. (2001). Residual transpiration rate, epicuticular wax load and leaf colour of pea plants in drought conditions. Influence on harvest index and canopy temperature. *Eur. J. Agron.* 15 57–70. 10.1016/S1161-0301(01)00094-6

[B87] Saneoka H. Ogata S. (1987). Relationship between water use efficiency and cuticular wax deposition in warm season forage crops grown under water deficit conditions. *Soil Sci. Plant Nutr.* 33 439–448. 10.1080/00380768.1987.10557590

[B88] Schreiber L. Riederer M. (1996). Ecophysiology of cuticular transpiration: comparative investigation of cuticular water permeability of plant species from different habitats. *Oecologia* 107 426–432. 10.1007/bf00333931 28307383

[B89] Seo P. J. Lee S. B. Suh M. C. Park M. J. Go Y. S. Park C. M. (2011). The MYB96 transcription factor regulates cuticular wax biosynthesis under drought conditions in *Arabidopsis*. *Plant Cell* 23 1138–1152. 10.1105/tpc.111.083485 21398568 PMC3082259

[B90] Seo P. J. Xiang F. Qiao M. Park J. Y. Lee Y. N. Kim S. G. (2009). The MYB96 transcription factor mediates abscisic acid signaling during drought stress response in Arabidopsis. *Plant Physiol.* 151 275–289. 10.1104/pp.109.144220 19625633 PMC2735973

[B91] Shao H. Chu L. Jaleel C. Zhao C. (2008). Water-deficit stress-induced anatomical changes in higher plants. *C. R. Biol.* 331 215–225. 10.1016/j.crvi.2008.01.002 18280987

[B92] Shepherd T. Wynne Griffiths D. (2006). The effects of stress on plant cuticular waxes. *New Phytol.* 171 469–499. 10.1111/j.1469-8137.2006.01826.x 16866954

[B93] Steinmüller D. Tevini M. (1985). Action of ultraviolet radiation (UV-B) upon cuticular waxes in some crop plants. *Planta* 164 557–564. 10.1007/BF00395975 24248232

[B94] Vogg G. Fischer S. Leide J. Emmanuel E. Jetter R. Levy A. A. (2004). Tomato fruit cuticular waxes and their effects on transpiration barrier properties: functional characterization of a mutant deficient in a very-long-chain fatty acid beta-ketoacyl-CoA synthase. *J. Exp. Bot.* 55 1401–1410. 10.1093/jxb/erh149 15133057

[B95] Wang H. Hao J. Chen X. Hao Z. Wang X. Lou Y. (2007). Overexpression of rice *WRKY89* enhances ultraviolet B tolerance and disease resistance in rice plants. *Plant Mol. Biol.* 65 799–815. 10.1007/s11103-007-9244-x 17960484

[B96] Wang M. Wang Y. Wu H. Xu J. Li T. Hegebarth D. (2016). Three TaFAR genes function in the biosynthesis of primary alcohols and the response to abiotic stresses in *Triticum aestivum*. *Sci. Rep.* 6:25008 10.1038/srep25008 PMC4845010 27112792

[B97] Wang W. Liu X. Gai X. Ren J. Liu X. Cai Y. (2015). *Cucumis sativus* l. wax2 plays a pivotal role in wax biosynthesis, influencing pollen fertility and plant biotic and abiotic stress responses. *Plant Cell Physiol.* 56 1339–1354. 10.1093/pcp/pcv052 26023108

[B98] Wang Y. Chen Z. Zhang B. Hills A. Blatt M. (2013). PYR/PYL/RCAR abscisic acid receptors regulate K+ and Cl- channels through reactive oxygen species-mediated activation of Ca2+ channels at the plasma membrane of intact Arabidopsis guard cells. *Plant Physiol.* 163 566–577. 10.1104/pp.113.219758 23899646 PMC3793038

[B99] Xia K. Ou X. Gao C. Tang H. Jia Y. Deng R. (2015). *OsWS1* involved in cuticular wax biosynthesis is regulated by Osa-mir1848. *Plant Cell Environ.* 38 2662–2673. 10.1111/pce.12576 26012744

[B100] Xu L. Zeisler V. Schreiber L. Gao J. Hu K. Wen J. (2017). Overexpression of the novel Arabidopsis gene *At5g02890* alters inflorescence stem wax composition and affects phytohormone homeostasis. *Front. Plant Sci.* 8:68 10.3389/fpls.2017.00068 PMC5266714 28184233

[B101] Xue Y. Xiao S. Kim J. Lung S. Chen L. Tanner J. (2014). *Arabidopsis* membrane-associated acyl-CoA-binding protein ACBP1 is involved in stem cuticle formation. *J. Exp. Bot.* 65 5473–5483. 10.1093/jxb/eru304 25053648 PMC4157719

[B102] Yeats T. H. Rose J. K. (2013). The formation and function of plant cuticles. *Plant Physiol.* 163 5–20. 10.1104/pp.113.222737 23893170 PMC3762664

[B103] Yu D. Ranathunge K. Huang H. Pei Z. Franke R. Schreiber L. (2008). Wax Crystal-Sparse Leaf1 encodes a beta-ketoacyl CoA synthase involved in biosynthesis of cuticular waxes on rice leaf. *Planta* 228 675–685. 10.1007/s00425-008-0770-9 18574592

[B104] Yu N. Sun Z. Huang X. Huang C. Guo Y. (2015). Variations of cuticular wax in mulberry trees and their effects on gas exchange and post-harvest water loss. *Acta Physiol. Plant.* 37 1–9. 10.1007/s11738-015-1856-1

[B105] Zhang J. (2011). China’s success in increasing per capita food production. *J. Exp. Bot.* 62 3707–3711. 10.1093/jxb/err132 21551079

[B106] Zhang J. Broeckling C. Blancaflor E. Sledge M. Sumner L. Wang Z. (2005). Overexpression of WXP1, a putative *Medicago truncatula* AP2 domain-containing transcription factor gene, increases cuticular wax accumulation and enhances drought tolerance in transgenic alfalfa (*Medicago sativa*). *Plant J.* 42 689–707. 10.1111/j.1365-313X.2005.02405.x 15918883

[B107] Zhang J. Broeckling C. Sumner L. Wang Z. (2007). Heterologous expression of two *Medicago truncatula* putative ERF transcription factor genes, *WXP1* and *WXP2*, in *Arabidopsis* led to increased leaf wax accumulation and improved drought tolerance, but differential response in freezing tolerance. *Plant Mol. Biol.* 64 265–278. 10.1007/s11103-007-9150-2 17347795

[B108] Zhang M. Jin Z.-Q. Zhao J. Zhang G. Wu F. (2015). Physiological and biochemical responses to drought stress in cultivated and Tibetan wild barley. *Plant Growth Regul.* 75 567–574. 10.1007/s10725-014-0022-x

[B109] Zhang Z. Wang W. Li W. (2013). Genetic interactions underlying the biosynthesis and inhibition of beta-diketones in wheat and their impact on glaucousness and cuticle permeability. *PLoS ONE* 8:e54129 10.1371/journal.pone.0054129 PMC3547958 23349804

[B110] Zhang Z. Wei W. Zhu H. Challa G. S. Bi C. Trick H. N. (2015). W3 is a new wax locus that is essential for biosynthesis of beta-diketone, development of glaucousness, and reduction of cuticle permeability in common wheat. *PLoS ONE* 10:e0140524. 10.1371/journal.pone.0140524 PMC4607432 26468648

[B111] Zhou L. Ni E. Yang J. Zhou H. Liang H. Li J. (2013). Rice OsGL1-6 is involved in leaf cuticular wax accumulation and drought resistance. *PLoS ONE* 8:e65139 10.1371/journal.pone.0065139 PMC3669293 23741473

[B112] Zhu L. Guo J. Zhu J. Zhou C. (2014). Enhanced expression of *EsWAX1* improves drought tolerance with increased accumulation of cuticular wax and ascorbic acid in transgenic *Arabidopsis*. *Plant Physiol. Biochem.* 75 24–35. 10.1016/j.plaphy.2013.11.028 24361507

[B113] Zhu X. Xiong L. (2013). Putative megaenzyme DWA1 plays essential roles in drought resistance by regulating stress-induced wax deposition in rice. *Proc. Natl. Acad. Sci. U.S.A.* 110 17790–17795. 10.1073/pnas.1316412110 24127586 PMC3816433

