# Supplementary material for: Molecular and Evolutionary Mechanisms of Cuticular Wax for Plant Drought Tolerance
Source: Front Plant Sci. 2017 Apr 28;8:621. doi: 10.3389/fpls.2017.00621 (PMC5408081; doi:10.3389/fpls.2017.00621)
Supplement: Supplementary file 1 [file Table_1.DOCX]

**Table S1** Number of published and predicted key cuticular wax genes involved in drought tolerance in different species.

| **Species/genes** | **WIN1/SHN1** | **CER1** | **CER6** | **DWA1** | **SHN2** | **SHN3** | **MYB94** | **MYB96** |
| --- | --- | --- | --- | --- | --- | --- | --- | --- |
| Arabidopsis locus | AT1G80600 | AT1G02205.3 | AT1G68530 | AT2G19430 | AT5G13320 | AT5G25390 | AT3G47600 | AT5G62470 |
| *Arabidopsis thaliana* | 1 | 1 | 1 | 1 | 1 | 1 | 1 | 1 |
| *Glycine max* | 2 | 5 | 4 | 3 | 1 | 3 | 4 | 0 |
| *Populus trichocarpa* | 2 | 4 | 3 | 1 | 0 | 0 | 1 | 0 |
| *Vitis vinifera* | 2 | 5 | 0 | 1 | 0 | 1 | 0 | 0 |
| *Oryza sativa* | 2 | 5 | 3 | 1 | 0 | 0 | 2 | 0 |
| *Hordeum vulgare* | 1 | 7 | 10 | 0 | 0 | 0 | 3 | 0 |
| *Picea abies* | 1 | 4 | 1 | 0 | 0 | 1 | 1 | 0 |
| *Selaginella moellendorffii* | 3 | 0 | 0 | 0 | 0 | 0 | 0 | 0 |
| *Physcomitrella patens* | 6 | 0 | 0 | 0 | 0 | 0 | 0 | 0 |
| *Marchantia polymorpha* | 1 | 0 | 0 | 1 | 0 | 0 | 0 | 0 |
| *Volvox carteri* | 1 | 0 | 0 | 0 | 0 | 0 | 0 | 0 |

Note: Candidate protein sequences were selected by BLASTP searches which satisﬁed E value <10^-10^ and query coverage >50%.
